# Evaluation of a developmental hierarchy for breast cancer cells to assess risk-based patient selection for targeted treatment

**DOI:** 10.1038/s41598-017-18834-5

**Published:** 2018-01-10

**Authors:** Sarah A. Bliss, Sunirmal Paul, Piotr W. Pobiarzyn, Seda Ayer, Garima Sinha, Saumya Pant, Holly Hilton, Neha Sharma, Maria F. Cunha, Daniel J. Engelberth, Steven J. Greco, Margarette Bryan, Magdalena J. Kucia, Sham S. Kakar, Mariusz Z. Ratajczak, Pranela Rameshwar

**Affiliations:** 1 Rutgers New Jersey Medical School, Newark, NJ USA; 2 Rutgers School of Graduate Studies at New Jersey Medical School, Newark, NJ USA; 3 Biocon Bristol Myers Squibb R&D Center, Bangalore, India; 4 PPD Laboratories, Wilmington, NC USA; 5 University of Louisville, Louisville, KY USA

## Abstract

This study proposes that a novel developmental hierarchy of breast cancer (BC) cells (BCCs) could predict treatment response and outcome. The continued challenge to treat BC requires stratification of BCCs into distinct subsets. This would provide insights on how BCCs evade treatment and adapt dormancy for decades. We selected three subsets, based on the relative expression of octamer-binding transcription factor 4 A (Oct4A) and then analysed each with Affymetrix gene chip. Oct4A is a stem cell gene and would separate subsets based on maturation. Data analyses and gene validation identified three membrane proteins, TMEM98, GPR64 and FAT4. BCCs from cell lines and blood from BC patients were analysed for these three membrane proteins by flow cytometry, along with known markers of cancer stem cells (CSCs), CD44, CD24 and Oct4, aldehyde dehydrogenase 1 (ALDH1) activity and telomere length. A novel working hierarchy of BCCs was established with the most immature subset as CSCs. This group was further subdivided into long- and short-term CSCs. Analyses of 20 post-treatment blood indicated that circulating CSCs and early BC progenitors may be associated with recurrence or early death. These results suggest that the novel hierarchy may predict treatment response and prognosis.

## Introduction

Despite improved treatments, breast cancer (BC) remains a clinical problem. BC cells (BCCs) can remain dormant for decades, commonly referred as cellular dormancy^1–8^. Cellular dormancy is a method by which the BCCs enter a state of cellular quiescence until it receives a que from the environment to proliferate^9^. Clinical outcome studies have documented disease re-occurrence from 1–20 years after initial treatment regardless of lymph node involvement^10^. There could be a long lag time between the initiation of the tumor to clinical diagnosis^11^. During this lag time, metastatic BCCs could escape into the circulation from undetectable, but developing tumor^8,12–14^.

The bone marrow (BM) can facilitate the survival of dormant BCCs for decades^15,16^. Thus, the BM is a significant organ when considering treatment for BC. Approximately 30% of BC patients have BM metastasis and about 50% of those may have cancer recurrence^17^. There is a strong correlation between BCCs in the BM and relapse. However, a direct evidence on cause-effect relationship between BCCs in the BM and metastatic recurrence requires additional studies. Regardless, it is evident that the presence of BCCs in the BM may be prognostic^8,18^. Thus, studies of BCCs using a developmental hierarchy as part of the characterization should be considered in future studies to correlate any association between developmental phenotype and outcome events including response. The stratification of BCCs into a robust hierarchy is missing in the literature. This study has begun to address this problem using gene chip arrays.

Metastasis can occur with < 0.1% of the BCCs entering the blood^19^. This percentage of BCCs that is linked to metastasis is similar to the frequency of cancer stem cells (CSCs) in tumor cell lines^3^. Since BCCs are heterogeneous, predicting which subset of BCCs will metastasize is difficult and hinders identification and stratification of BCCs hierarchically. Stratification would provide insight on the tissue microenvironment (TME) and how the TME influences drug resistance to therapy and immune responses^20,21^. A hierarchical stratification of BCCs could allow for precise targeting of BCCs in organs such as the BM. Presently, the BM poses a major challenge to acquire effective treatment to target BCCs that require targeting the milieu of immune suppressor cells such as mesenchymal stem cells in the BM^3,4,22^. Treatment will need to overcome the ability of endogenous BM cells to sustain quiescence of BCCs^3,4,23–29^. Specifically, cells of the BM niche can retain BCCs in a dormant phase, making them difficult to treat. In addition, it is important to note that any treatment of BCCs within the BM microenvironment must avoid overt toxicity to the endogenous hematopoietic stem cells^27,30,31^.

There is a growing acceptance among the scientific community that new treatments are needed to target CSCs since this will remove the initiating cells and halt the propagation of the tumor^32–34^. The premise underlying this strategy is that the loss of the initiating tumor cells will cause the bulk cancer to regress. However, this strategy needs to consider the possibility that the non-CSCs/cancer progenitors may dedifferentiate into CSCs^21,35,36^. We address these problems by developing a hierarchy of BCCs since this would be needed to study if dedifferentiation occurs, and if so, identify the ease by which the particular BCC subset can dedifferentiate. We identified three new membrane proteins, GPR64, TMEM98, FAT4 using the Affymetrix data analyses^37,38^. These membrane proteins, along with other markers reported for CSCs, were used to establish a hierarchy of BCCs. The developed hierarchy was tested with blood samples from BC patients that were obtained after treatment. The circulating BCCs were then associated with the patients’ outcome up to two years after treatment.

## Results

### Affymetrix Gene Array Analyses/Differential Expression

**Figure 1:**
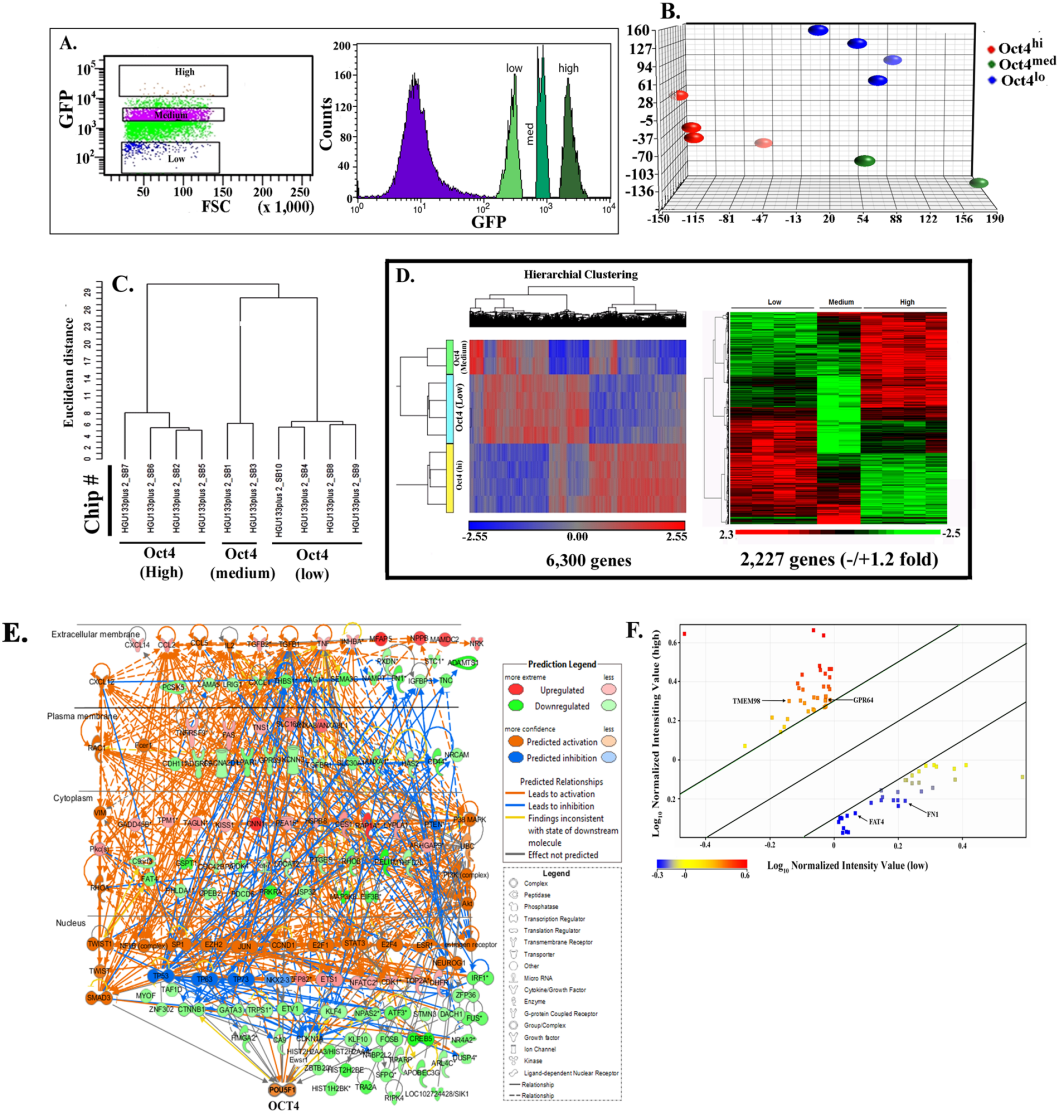
Selection of samples for Affymetrix Genechip arrays with validation of groups. (**A**) A scattergram used to select different subsets from MDA-MB-231 cells, stably transfected with pEGFP1-Oct3/4 (left panel). The relative GFP intensity of the three subsets are also depicted in a histogram (right panel). (**B**) PCA generated from Partek analysis, showing the distribution of conditions and clustering of sample replicates. (**C**) Hierarchical clustering of the samples using 2227 genes differentially expressed at p < 0.001 and FDR < 1.12%. (**D**) Heat map with hierarchal clustering generated from Partek compares all three conditions and represents 6300 genes differentially expressed (1.2+/−fold) at *p* < 0.1 and 10% FDR (false discovery rate). Two-sample T-test or F-test (more than 2 classes) based *p* value was calculated for each significant gene with random variance model. (**E**) Molecular interaction network of BC overrepresented genes associated with OCT4-expressing BCCs generated from the information in the Ingenuity knowledge base. Top upstream regulators, identified by IPA core analysis, were incorporated in the network. Molecule Activity Predictor (MAP) illustrates upstream/downstream activation or inhibition of molecules in the network. Solid lines represent direct relationship and dashed line represents indirect relationship between nodes. Prediction legend and gene product’s functional class are shown in the legend key. **(F)** A scatter plot developed in Genespring using normalized signal values in Log_10_ scale. The statistical significance was assessed with an unpaired *t*-test between Oct4^hi^ and Oct4^low^, cut-off points were *p*-value < 0.05 and fold-change > 2.0. Specific genes of interest have been annotated on the plot.

As the first step to stratify BCCs, we performed global gene expression analyses using MDA-MB-231. The method used to select three different subsets was previously described^3^, and based on the relative GFP intensities of cells stably transfected with the Oct4A promoter linked to GFP. Based on previous studies, we showed a direct correlation between Oct4A and GFP intensity^3^. Despite previous studies showing low levels of Oct4 protein maintaining pluripotency^39^, in our model, which is based on previous report, functional and biochemical studies indicated that the highest expression of Oct4A is linked to ‘stemness’ of BCCs^3^. We should also note that most of the Oct4 pseudogenes are not translated to proteins. Thus, for the purpose of simplifying our model, we have designated relative GFP intensities with Oct4A promoter as Oct4^hi^ (5%), Oct4^med^ (30%) and Oct4^low^ (10%)^3,26^ (Fig. 1A). We then performed global gene expression analyses with total RNA from four different sets of MDA.MB-231, using the Affymetrix Genechip Human Genome U133 Plus 2.0 arrays. Oct4^hi^ and Oct4^low^ were analyzed in quadruplicates and Oct4^med^, in duplicate (Fig. S1). The data were analyzed with multiple database and software tools, Partek^®^ Genomics Suite software, Gene Spring GX 10.0.2, QIAGEN’s Ingenuity® iReport, and BRB-Array Tools.

We conducted principal component analyses (PCA) to determine the similarities and differences among array results of each sample. The results indicated confidence with respect to similarities as indicated by clustering of the replicates from each BCC subset (Fig. 1B). Additionally, the clusters were far apart, indicating distinct separation of the overall gene expression pattern among the subsets (Fig. 1B).

BRB-Array Tools provided a multi-dimensional scaling analysis, supporting distinct gene expression among the samples and depicted the tight grouping of replicate sample and greater separation among the BCC subsets (Fig. S2). The similarities and differences were obvious in the hierarchical clustering of the samples linking Oct4^med^ and Oct4^lo^ (Fig. 1C). Both subsets were linked to Oct4^hi^ BCCs, further supporting a hierarchy among the three Oct4A subsets.

Heat maps generated with Partek and BRB-Array Tools incorporated the differentially expressed genes. The map with 6,300 genes showed clustering among the replicates, but with distinct gene intensities among the three BCC subsets (Fig. 1D, left). A hierarchical clustering of genes with −/+1.2 fold changes (2,227 genes) showed distinct pattern of gene expression among the three subsets (Fig. 1D, right). The results showed distinct global gene expression among BC subsets.

The analyses programs could not incorporate the data for Oct4^med^ BCCs due insufficient replicates. Thus, we compared Oct4^hi^ and Oct4^lo^ BCCs for differentially expressed genes using the normalized signal values. Those at higher statistical relevance are highlighted (*p* value, < 0.05; fold change > 2.0) (Fig. 1E, Table S1).

### Biological networks analysis/canonical pathways

The IPA knowledge base compared genes with >1.5 fold for those associated with breast and ovarian cancers, and then determined the connections to Oct4/POU5F1 (Fig. 1F). Molecule Activity Predictor illustrates activation or inhibition of regulators/molecules in the networks. In Oct4^hi^ BCCs, Oct4/POU5F1 expression was highly activated including its 34 transcriptional activators. Activation used in the network’s output implies functional activation and increase in the gene expression. High expression of Oct4 showed upregulation/activation of genes linked to TNFα and TGFβ signaling, TGFB1, TGFB2, INHBA, SMAD3 and SMAD4, suggesting that Oct4 may be linked to microenvironmental functions. In general, the tumor cells are within a niche of cytokines. In Oct4^med^ BCCs, TNFα was inhibited and TGFβ signaling molecules: either downregulated (TGFB2) or poorly activated (TGFB1, TGFBR2, SMAD3 and SMAD4). Interleukins (IL-1, IL-2 and IL-4) and the proinflammatory CCL2 were highly activated in SNAI1, SNAI2, TWIST1, ZEB1 BCCs. The key molecules linked to epithelial mesenchymal transition (EMT), SNAI1, SNAI2, TWIST1, ZEB1, CXCL12, VIM, CDH2, MMP2, CXCR4, ERBB2, RAC1, Notch, PARD6A and PARD6G, were highly activated whereas E-cadherin (CDH1) and connexin-43 (GJA1) were inhibited. We also analyzed the data set for membrane-spanning genes, which is the focus of this paper. Our goal was to use the dataset to identify novel genes that can stratify BCCs.

### Selection of markers in the gene set

**Figure 2:**
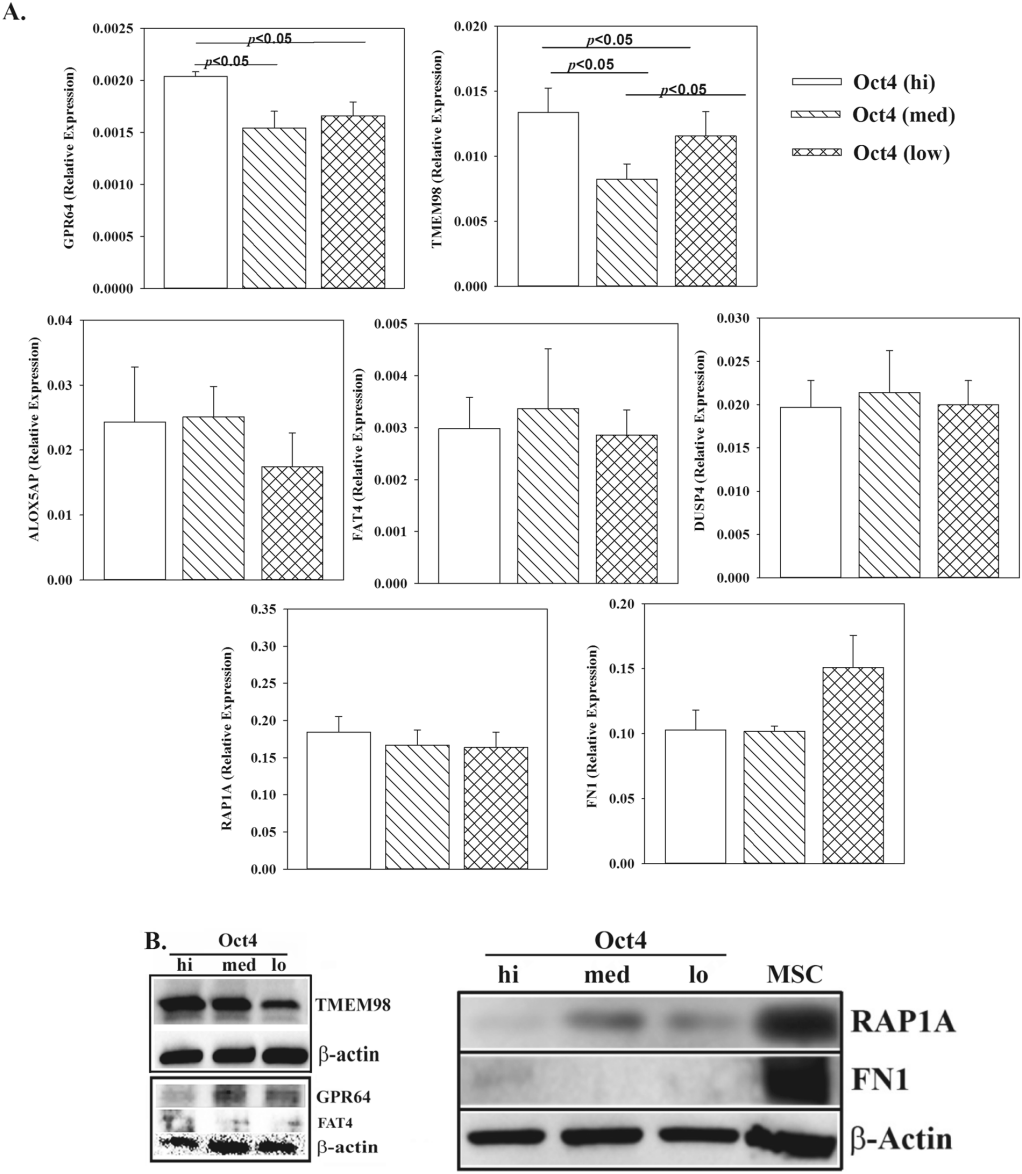
Expression of selected genes in BCC subsets. (**A**) Real time PCR for GPR64, FAT4, TMEM98, ALOX5 AP, DUSP4, RAP1A and FN1. The RNA was isolated from Oct4^hi^, Oct^med^ and Oct4^low^ subsets from MDA-MB-231 stably transfected with pEGFP1-Oct3/4. The results are shown as relative to β-actin expression with mean ± SD for four independent experiments. (**B**) Western blots for GPR64, TMEM98, FAT4, RAP1A and FN1 using whole cell extracts expression from Oct4^hi^, Oct^med^ and Oct4^low^, isolated as for ‘A’. The results represent four different independent experiments.

The differentially expressed genes were validated by real-time PCR. We showed the results for a few, in particular those with immediate interest since we sought membrane proteins for the stratification of BCCs; hence the selection of FAT4, TMEM98 and GPR64 (Fig. 2A). The results for these three genes from the arrays were validated by western blot (Figs 2B and S3). RAP1A, which showed the highest difference in the Affymetrix data could not be validated by PCR or western blot and was not selected for further studies. Fibronectin (FN) was studied since as a control since it is an EMT marker and is therefore expected in the Oct4^hi^ subset, which has been shown to be the CSC^3^. In summary, the findings validated FAT4, TMEM98 and GPR64 as key membrane proteins for further development of a hierarchical rank among BCC subsets.

### ALDH1 and Telomere Length in BCC subsets

**Figure 3:**
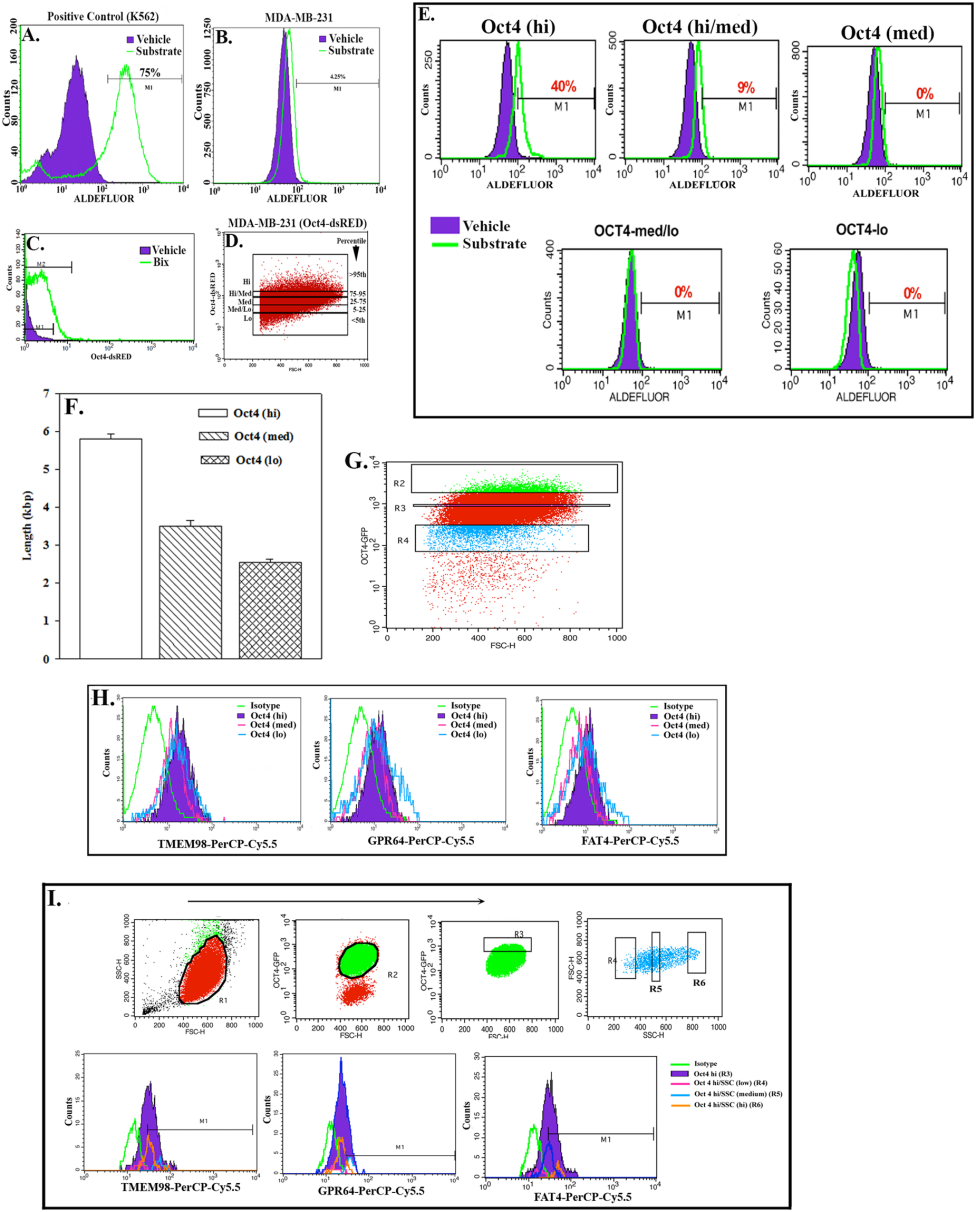
Characterization of BCC subsets. ALDH1 activity was assessed in positive control with K562 cells (**A)** and experimentally, with heterogeneous MDA-MB-231 cells (**B**). The presence of Oct4-dsRED-SB in MBA-MB-231 cells was determined by Bix treatment in flow cytometry (**C**). Different subsets of Oct4-dsRED-SB-transfected MDA-MB-231 cells (**D**) were assessed for ALDH1 (**E**). Similar sorting of three MDA-MB-231 subsets were analyzed for telomere length using the TeloTAGG kit (**F**). MDA-MB-231, stably transfected with Oct4-GFP (left panel) was labeled with PE-anti-TMEM-98, -GPR64 and -FAT4 and analyzed by flow cytometry based on the relative GFP levels (n = 5; **G** and **H**). Further assessment for TMEM-98, GPR64 and FAT4 within Oct4^hi^ BCCs was based on size. Oct-4-GFP transfected MDA-MB-231 cells were gated based on GFP expression (**I**, top second panel) followed by gating of the 5% of cells with the highest GFP (**I**, R3, top third panel-Oct4^hi^). R3 was divided based on side (SSC) and forward scatter (FSC) (R4-R6) and then analyzed for TMEM98, GPR64 and FAT4 (**I**, lower panels). The results represent four independent experiments.

CSCs have been reported to be within the Oct4^hi^ BCC subset^3^. Since ALDH1 has been reported in CSCs (38), we examine the different BCCs for ALDH1. Positive control with K562 cells showed a major shift to the right (Fig. 3A). MDA-MB-231 cells showed 4.25% (+) for ALDH1 (Fig. 3B). The observation was consistent with BCCs comprise of ~5% CSCs (Fig. 3B)^3^. Next, we stratified BCCs, based on the relative intensities of fluorescence in cells stably transfected with Oct4-dsRED-SB. These analyses could not use Oct4-GFP due to the overlap with the ALDH1 substrate on the FACScan. BCCs were stably transfected with pOct4-dsRED-SB. Since we used this vector for the first time, we validated its function by treating the transfectants with Bix^3^. The purpose of using Bix is to inhibit methytransferases and deacetylation of the Oct4 regulatory region within pOct4-dsRED in all BCC subsets, regardless of endogenous expression^40^. The results with Bix indicated that pOct4-dsRED was functional (Fig. 3C). Using the gating scheme in Fig. 3D, 40% ALDH1(+) was observed in Oct4^hi^, 9% in Oct4^hi/med^ and no detection in the Oct4A reduced BCC subsets (Fig. 3E).

High telomerase generally correlates with increase in the telomere length^41^. We therefore examined Oct4^hi^, Oct4^med^ and Oct4^low^ BCCs for telomere length. The results showed a direct correlation between Oct4 levels and telomere length (Fig. 3F).

### TMEM98, GPR64 and FAT4 in BCC subsets

BCCs with stable pOct4-GFP were studied for TMEM98, GPR64 or FAT4 by flow cytometry. Gating the MDA-MB-231 and T47D based on Oct4A expression (Fig. 3G), we observed similar results. Thus, representative histograms for MDA-MB-231 indicated TMEM98, Oct4^hi^ > Oct4^low^ > Oct4^med^; GPR64 and FAT4, Oct4^low^ > Oct4^hi^ > Oct4^med^ (Fig. 3H). The results indicated distinct expressions for TMEM98, FAT4 and GPR64 among the BCC subsets.

ALDH1 was detected in 40% of Oct4^hi^ BCCs (Fig. 3E). Since this subset also contained tumor initiating cells, we proposed that Oct4^hi^ BCCs might be heterogeneous^3^. Thus, we analyzed Oct4^hi^ BCCs for FAT4, TMEM98 and GPR64, based on scatter pattern. The Oct4^hi^ BCCs (Fig. 3I, R3) were stratified by side (SSC) and forward scatter (FSC) (Fig. 3I, R4-R6) and then analyzed for FAT4, TMEM 98 and GPR64. The results confirmed heterogeneity within the Oct4^hi^ BCCs with respect to the three markers (Fig. 3I, lower panels).

### Distribution of TMEM98, GPR64 and FAT4 within CD44+/CD24− BCCs

**Figure 4:**
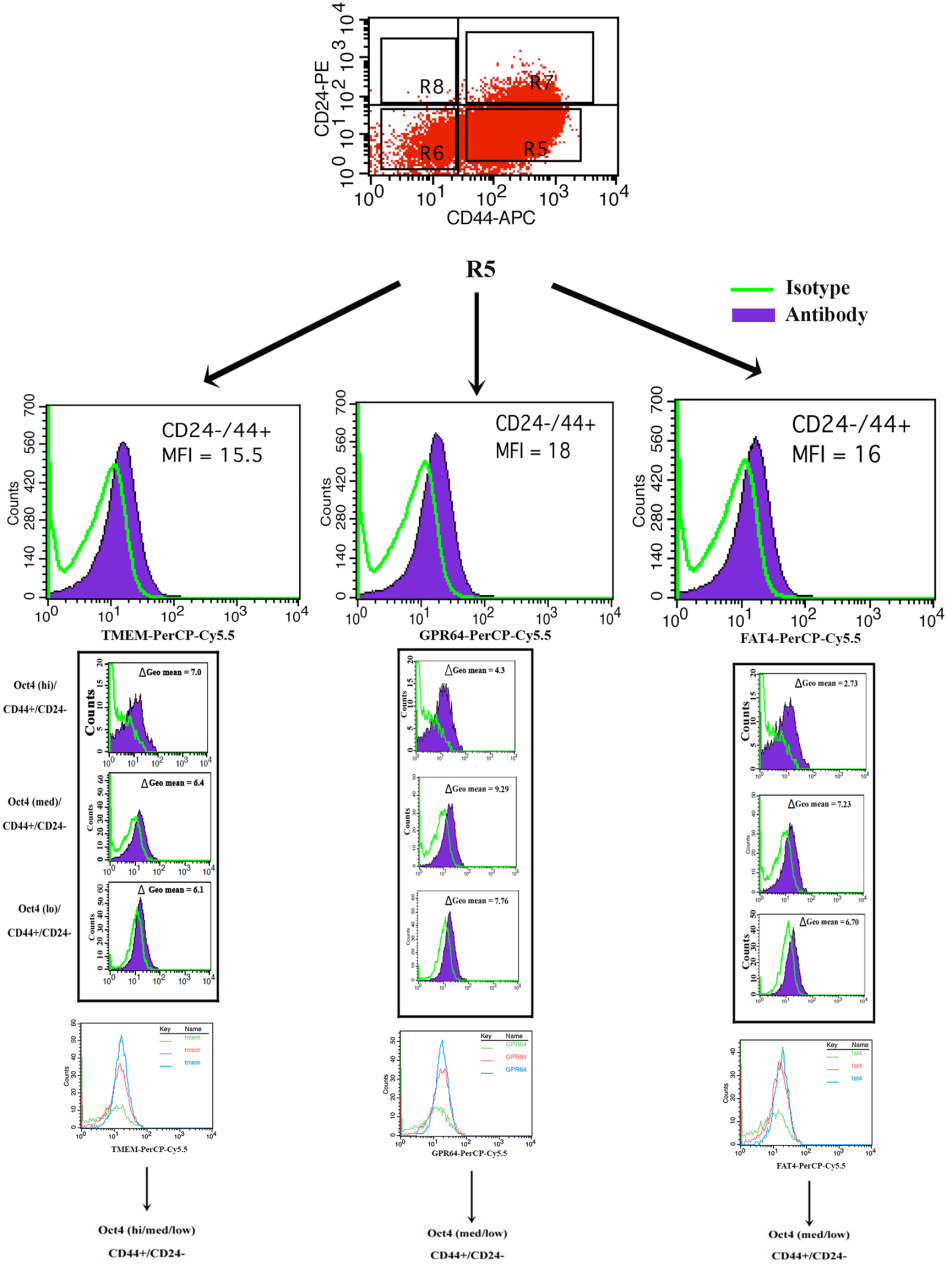
Co-expressions of TMEM98, GPR64 and FAT4 in the CD44+/CD24− BCC subset. BCCs, stably transfected with Oct4-GFP, were co-labeled with CD44-APC and CD24−PE and the population of CD44 + /CD24− (Top panels) further studied for the expression of TMEM98, GPR64 and FAT4 (middle panels). CD44+/CD24− populations were further stratified based on Oct4 expression (Oct4^hi^, Oct4^med^ and Oct4^low^) and then studied for TMEM98, GPR64 and FAT4 (lower panels). The Δ Geometic (Geo) mean fluorescence intensity (MFI) for TMEM98, GPR64 and FAT4 are shown in each panel. The Geo mean was calculated as the change from the actual MFI – the MFI of the isotype.

CD44 + CD24− BCCs, which were reported to be markers of CSCs, needs further hierarchical stratification as this subset was shown to be heterogeneous with respect to CSCs^3,38^. We therefore studied how the CD44 + CD24− BCCs can be further stratified based on TMEM, GPR64 and FAT4 expressions. We observed a shift to the right for each marker, indicating their expression (Fig. 4/middle panels). Next we analyzed CD44 + CD24− BCCs based on Oct4 (hi, medium, low) for TMEM98, GPR64 and FAT4. The geometric (Geo) mean for TMEM98 showed its presence on Oct4 (hi/med/lo) and, GRP64 and FAT4 on Oct4 (med/lo) CD44 + CD24− BCCs.

### Assembling BCC hierarchy

**Figure 5:**
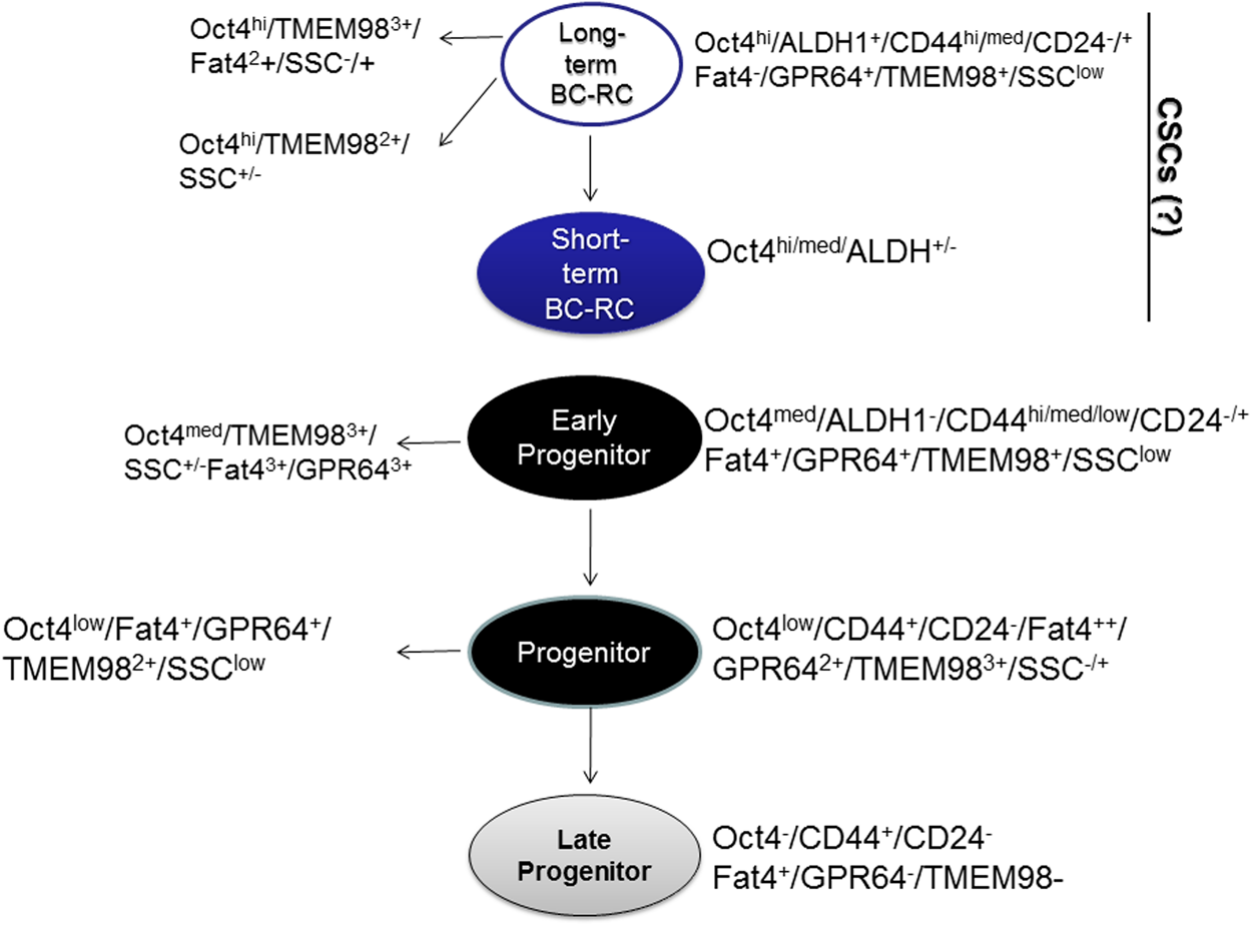
Working hierarchy of BCCs. The hierarchy was based on the analyses of BCCs depending on Oct4A expression, previously reported^3^. The present studies are based on the use of additional antibodies, identified in the gene chip arrays (FAT4, TMEM98 and GPR64). The studies were done in combination of known markers of CSCs: ALDH1, CD24 and CD44.

The methods used for cell lines, analyses of ALDH1 used to assemble the hierarchy of BCCs are summarized in a flow diagram (Fig. S4). Although Oct4^hi^ BCCs were shown to contain CSCs^3^, we only found 40% ALDH1+ with 9% at Oct4^hi^ and Oct4^med^ interface (Oct4^hi/med^) (Fig. 3E). We assigned the Oct4^hi/med^ BCCs with reduced Oct4A-GFP intensity as short-term BC repopulating cells (BC-RC) and assigned Oct4^hi^ subset with the highest GFP intensity as term long-term BC-RC (Fig. 5). We branched Oct4^hi^ BR-RC based on the side scatter (SSC) for FAT4, GPR64 and TMEM98 (Fig. 3I, 5/upper left).

Oct4^med^ BCCs with further stratification of GFP intensities formed branches from the early BC progenitors (left ‘branches’) (Fig. 5, third circle). Similar analyses established Oct4^low^ BCCs (Fig. 5, 4^th^ circle). Oct4^low^ and Oct4- BCCs, based on FAT4. GPR64 and TMEM98, divided these subsets into progenitors and ‘late progenitors’ (Fig. 5). CD44 + /CD24− BCCs were demarcated due to varied Oct4A and FAT4, TMEM98 and GPR64 (Fig. 4) for inclusion into the hierarchy.

### Identifying circulating tumor cells in BC patients

We investigated if the developed hierarchy can predict treatment response and prognosis in 20 BC patients (Table 1). Due to ethical reasons, we studied time points, mostly after treatment using blood remaining after clinical tests. Table 1 describes the demographics and disease course of the patients. Flow cytometry with markers shown in the hierarchy (Fig. 5) assigned the developmental stage of BCCs for the patients (Table 2). The method used to analyze the patients’ samples were similar to analyses of cell lines as outlined in Fig. S4. Subject 5 showed high amounts of CSCs before treatment and early BC progenitors after treatment. At the time of writing (6 months post-treatment), this subject was diagnosed with bone metastasis. Similarly, subject 13 ended treatment with circulating CSCs and shortly after was diagnosed with bone pain. The preliminary studies indicated that the markers in the hierarchy may predict BC outcome and treatment response.

**Table 1:** Patient demographics.

Patient	Age (Yrs)	Ethnicity	Diagnosis	Clinical Disease at Blood Sampling	Treatment
1	50	Black	Relapse ER+PR+Her2−	Yes	Hormone
2	35	Hispanic	Relapse ER-PR-Her2−	Yes	Chemotherapy
3		Black	Relapse ER-PR-Her2−	Yes	Chemotherapy
4	60	Black	Stage II	No	Adjuvant: Doxorubicin, cyclophosphamide
5 A	40–49	Caucasian	Relapse ER+PR+Her2−	Yes	Pre-Chemotherapy (Palbociclib)
5B	↓	↓	↓	No	Post-Chemotherapy
6	63	Caucasian	Benign (needle biopsy)	No	None
7	58	Hispanic	ER-PR-Her2−Stage I	No	Lumpectomy; radiation; chemotherapy (Paclitaxel and Carboplatin)
8	54	Caucasian	ER-PR-Her2−Stage II	No	Surgery (mastectomy), Chemotherapy
9	36	Black	ER+PR+Her2+	No	Post-surgery, radiation, Herceptin; Chemotherapy (AC → T); On Tamoxifen
10	53	Hispanic	Stage II ER+PR+Her2+	No	Lumpectomy; 4 cycles Paclitaxel; One year Herceptin; 4 yrs Tamoxifen
11	56	Hispanic	Stage III ER+PR-Her2+	No	Mastectomy; Radiation; Chemotherapy (Docetaxel, Paclitaxel, Herceptin)
12	56	Hispanic	Stage I ER+PR-Her2−	No	Mastectomy; Taxol, No radiation, On Tamoxifen
13	66	Black	Stage IV ER+PR+Her2+	Yes	Taxotere; Herceptin, Hormone (Faslodex) Post-chemotherapy – bone pain
14	64	Hispanic	Stage I ER+PR+Her2+	No	ECOG 5103 Trial (Bevacizumab), Herceptin, Hormone (Arimidex)
15	59	Black	ER+PR+Her2− Stage II	No	Lumpectomy, radiation, chemotherapy (AC → T); Hormone (Tamoxifen)
16	50	Caucasian	Stage III ER+PR+Her2+	No	Post-treatment (Chemotherapy and surgery)
17	48	Hispanic	Stage III ER-PR-Her2−	No	Post-treatment (2015); follow-up
18	45	Black	Stage II (2006); Relapse (2015) ER-PR- Her2−	Chest wall (2015)	Mastectomy at initial diagnosis (2006); Radiation in 2015
19	48	Hispanic	Stage IIA ER+PR+Her2+	No	Mastectomy; Completed Chemotherapy (Adriamycin, Cytoxan, Paclitaxel); Hormone (Tamoxifen)_
20	85	Black	Stage IV ER+PR+Her2−	Yes	Hormone (Arimidex)

AC → T = Adriamycin, cyclophosphamide, Paclitaxel.

Pre- indicates that the blood was analyzed before chemotherapy.

Post- refers to blood sampling after chemotherapy or after surgery.

In cases where ‘Relapse’ is not included in Column 4, this indicates that the tumor was primary and not from relapse.

**Table 2:** Phenotype of circulating BCCs in patients.

Patient	Treatment Pre Ongoing Post	Date Tested	Circulating BBCs	Outcome (3/2017)
1	+	3/10/15	Early Progenitor	Metastasis
2	+	3/10/15	Late Progenitor	Passed
3	+	3/12/15	Early Progenitor	Passed
4	+	3/18/15	Early Progenitor	No clinical active disease
5A	+	4/29/15	CSC+++	Not applicable
5B	+	7/8/15	Early Progenitor	Bone metastasis
6	None	5/5/15	ND	No clinical active disease
7	+	10/12/16	ND	No clinical active disease
8	+	10/12/16	ND	No clinical active disease
9	+	8/11/16	Late Progenitors	No clinical active disease
10	+	8/11/16	Late Progenitors	No clinical active disease
11	+	8/15/16	ND	No clinical active disease
12	+	8/16/16	ND	No clinical active disease
13	+	8/17/16	CSC++	Bone Metastasis
14	+	8/18/16	ND	
15	+	10/12/16	Early Progenitor	No sample post-treatment
16	+	5/3/16	ND	No clinical active disease
17	+	8/30/16	ND	No clinical active disease
18	+	9/6/16	Progenitor	No clinical active disease
19	+	9/7/16	Mixed Progenitors (Early-Late)	No clinical active disease
20	+	9/15/16	ND	No clinical active disease

The patient demographics shown in Table 1 depicts the stratification of breast cancer cells detected in the peripheral blood during and after treatment.

ND: No breast cancer detected; CSC: Cancer stem cells, based on the hierarchy shown in Fig. 5.

## Discussion

We report on the genetic and phenotypic differences in BCC subsets with a working hierarchy. The population with high Oct4A showed heterogeneity depending on the scatter pattern and CD44/CD24 expression (Fig. 5). ALDH1, which is generally linked to stem cells was incorporated to demarcate the most primitive BCCs into more and less mature cells, with the possibility of varied multipotency (Fig. 5).

We applied the hierarchy (Fig. 5) by testing patients’ blood during and after treatment (Tables 1 and 2). The analyses were done with limited amount of blood since the samples were those remaining after clinical diagnosis. The data indicated that the hierarchy may be relevant to treatment response and long-term outcome. The study lacked a timeline change due to the ethics of drawing blood for research when the patients were on chemotherapy. Subject (#5) was assessed before and after treatment since she was not anemic and donated additional blood samples. As expected Subject 5, who was a relapsed patient started with high CSCs and ended with early progenitors. After 6 months, this patient was presented with bone metastasis. The information provided with the blood samples suggested the hierarchy may aid be relevant to precise treatment for BC. Residual BCCs at the lower end of the hierarchy may predict a good outcome, perhaps due to their inability to initiate a tumor. This may contrast residual BCCs in the upper hierarchy, which could indicate poor prognosis. Also, BCCs the more primitive residual cells may dedifferentiate into CSCs.

Despite the reproducibility among the replicates in the arrays, some of the changes could not be validated by real time PCR. This may be explained by the RMA normalization method, which entails averaging the intensity of all probes. Since we were interested in selecting membrane proteins for clinical application, we focused on those genes then selected TMEM, GPR64 and FAT4, which contributed to an expanded BCC hierarchy^3^.

CD44 + /CD24− BCCs showed varied expressions of TMEM98, GPR64 and FAT4 in subsets based on Oct4A levels (Fig. 4). These findings suggested that CD44 + /CD24− BCCs while encompassing CSCs, could be heterogeneous. The identification of surface markers to stratify BCCs is important for the isolation of primary subsets thereby avoiding *in vitro* changes of the primary tumor. The hierarchy developed from this study can be used to select primary BCC subsets as would be required for precise analyses in cancer biology.

FN in Oct4^hi^ BCCs was highly significant due to its link to epithelial-mesenchymal-transition. Since the CSCs can adapt dormancy, the production of FN1 within the stromal compartment could be an indicator that the dormant BCCs have become part of the normal hematopoietic microenvironment, ensuring their survival. In BM, the dormant BCCs will need to survive within a complex system of hematopoiesis, and FN1 is part of the BM stromal microenvironment.

The established a network with the microarray data explained how Oct4 (POU5F1) was linked to the validated genes (Fig. S5). Since Oct4 expression was used to isolate the different BCC subsets, the link between Oct4 and other genes provided insights into how this stem cell-associated gene Oct4, which can demarcate different subsets, is linked to developmental stages of BCCs, and the three membrane markers, TMEM98, FAT4 and GPR64. Not a surprising link, Oct4 connected to Twist, which is gene associated with EMT^42^. Similar links to Oct4 was shown for additional markers in Fig. 1E. The link among genes in the Oct4^hi^ BCCs, shown to comprise the CSC subset, is relevant to an understanding of CSC survival, and the development of targeted treatment^3,43^.

Despite the lack of reliable markers of CSCs, this study has begun to show several potential markers. It is yet to determine if the newly identified markers can identify BCCs in all microenvironments, or if the markers might be involved in the behavior of the BCCs within a specific organ. EpCAM, reported on CSCs, showed a 1.1 fold change in our array^44^. In our hierarchy, we identified the Oct4^hi^ BCCs, which were previously shown to be CSCs^3^. Similarly, it is possible that EpCAM-expressing BCCs may require further stratification. Also, the variations among antibodies will require robust validation to translate the findings to patients. As suggested for EpCAM^44,45^, the markers shown in this study may be important for follow-up studies to target using the chimeric antigen receptor approach.

The hierarchy shown in Fig. 5 were generated based on the relative intensities of Oct4-GFP/RFP in BCCs. The other genes linked to these broad subsets, if used to isolate the respective BCC subsets could allow for functional information, in addition to the discussed utility for prognosis. Oct4 is shown to link with genes that have been reported to have significant roles in tumor progression, such as EMT markers (Fig. 1F). IPA analyses with Oct4 gene as the focus indicated important molecules in BC progression and at the same time, displayed how these molecules are connected, their expression levels, and sub-cellular localizations. Our analyses added upstream regulators of Oct4. In summary, this study has just begun to determine how the Oct4 gene is linked to gene expressions and function. Together the data can be combined for long-term outcome studies, and to sort subsets of BCCs for functional studies. The telomere length shown in this study begins to examine functional studies. We anticipate to isolate primary BCC subsets to expand on these studies, in addition to other functional analyses.

The biological replicates presented in this study used one cell line. The data seem to fit the triple positive BCCs since T47D cells were used in the flow cytometry studies. The hierarchy developed from these studies were applied to patients with phenotypically different tumors, with respect to hormone receptor expression. Thus, although the limitation in the initial studies is the lack of analyses with T47D, the data provide information to expand the clinical analyses in a large cohort of patients.

This paragraph discusses the translational relevance of the study. The stratification of BC developmental subsets for clinical translational purposes is a major hurdle. The consequence being the lack of clarity to determine the cancer subsets being targeted. If the relative maturity of the surviving BCCs can be identified, this may provide insights on the patient outcome, such as time to cancer re-occurrence. We applied genomic data from Affymetrix analyses to develop a hierarchy (working) in which the least mature BCCs are placed at the top. The working hierarchy forms the basis for future studies to expand the relative maturity of BCCs. The hierarchy incorporated TMEM98, GPR64 and FAT4, CD44, CD24, ALDH1 and Oct4A. The significance of the hierarchy was evaluated by analyzing the blood of patients. Due to ethical constraints, we mostly collected blood samples after treatment to evaluate what specific cancer subset remained. The patients’ outcome was monitored up to 1–2 years after the last treatment, which coincided with the blood testing. Although 24 blood samples were collected, we could only test 20 due to technical difficulties. Despite the lack of a larger study with statistical analyses, the current data strongly suggested that the hierarchy could predict treatment response and outcome. A larger multi-center control trials could lead to the development of precise treatment for breast cancer patients.

The final paragraph discusses the limitation of the study and provide a plan for future studies. We previously performed functional assay to show that Oct4^hi^ BCCs comprise the tumor initiating cells^3^. The present study used phenotypic studies and apply the findings to test clinical samples from BC patients. However, the hierarchy shown in Fig. 5 requires functional studies using *in vivo* models. We are in the process of generating antibodies to the extracellular region of GPR64, FAT4 and TMEM98. The antibodies will be used to select subsets of BCCs from cell lines and primary tumor cells for functional studies. A prospective study is needed to validate the prognostic significance of developmental markers identified here, and use a developmental hierarchy-based patient selection in enabling precise treatment approaches.

## Materials and Methods

### Reagents

DMEM, RPMI 1640, α-MEM, recombinant human insulin were purchased from Life Technology (Grand Island, NY), fetal calf sera (premium) from Hyclone Laboratories (Logan, UT), restore western blot stripping buffer, cytoplasmic extraction kit and SuperSignal West Femto Maximum Sensitivity Substrate reagent from Thermo Scientific (Rockford IL), effectene transfection reagent from QIAGEN (Valencia, CA).

### Antibodies

Rabbit anti-human TMEM98 (Catalog #NBP1-84154), FAT4 (Catalog #NBP1-78381) and GPR64 (Catalog #NBP1-84906) were purchased from Novus (Littleton, CO), RAP1A, APC- mouse anti-human CD44 from BD Pharmingen (San Jose, CA), PE-mouse anti-human CD24 and PE-murine IgG isotype from Biolegend (San Diego, CA), PerCP-Cy5.5- anti-rabbit IgG from Santa Cruz Biotechnology (Dallas, Tx), PE-anti- human pan-cytokeratin from Abcam (Cambridge, MA) and APC-mouse IgG isotype control from (BD Biosciences). Mouse anti-fibronectin (FN1) was prepared as ascites from a hybridoma cell line that was purchased from ATCC.

### Cells

MDA-MB-231 (highly invasive, basal-like) and T47D (low-invasive, luminal) BCCs and K562 cells were purchased from American Type Culture Collection (ATCC). The cells were cultured as per ATCC instructions.

BCCs, stably transfected with Oct4-GFP, were previously described^3^. Briefly, the cells were transfected with pEGFP1-Oct3/4 and then selected with neomycin. Similarly, stable transfectants of BCCs with pDsRed2-1-Oct3/4-SB were selected with neomycin.

### Ethical statement

The use of human blood was approved by the Institutional Review Board (IRB) of Rutgers, Newark Campus. All subjects signed the informed consent forms. Furthermore, the studies were conducted as outlined in the IRB protocol.

### Human subjects

Blood (2–5 mL) was taken from left over samples after diagnostic tests from BC patients. The demographics of the patients and their treatment at the time of blood draw are outlined in Table 1.

### Vectors

pEGFP1-Oct3/4, which contained the Oct4A promoter, was generously provided by Dr. Wei Cui (Imperial College London, UK). The insert *Oct4* insert from pEGFP1-Oct3/4 was removed using the restriction enzymes, *Hind*III and *Age*I. Since the insert and the vector were similar size, a further digestion was performed with *Not*I to cut the vector backbone from pEGFP. This separated the backbone vector and the *Oct4* insert. The excised insert was ligated into pDsRed2-1. The new vector containing the Oct4A promoter was designated, pDsRed2-1-Oct3/4-SB.

### Induced *Oct4* expression

Induction of Oct4 was performed as described^3^. BIX-01924 was added at varying concentrations (0 nM, 10 nM, 100 nm, 1 μM, 3 μM, and 10 μM) to 6-well tissue culture plates (Falcon, Pittsburg, PA). Induced *Oct4* was indicated by the expression of dsRed, after 24 h, by flow cytometry.

### Sorting of BCC subsets

BCCs were stably transfected with pEGFP1-Oct3/4 and then profiled based on GFP intensity. The relative intensity formed the basis for sorting with the fluorescent-activated cell sorting (FACS) with a FACSAria II cell sorter (BD Biosciences). The sorting was performed as previously described, using the top and lower 5% GFP cells and the population in the middle of the two extreme subsets (3). The sorted cells were designated Oct4^hi^, Oct4^med^ and Oct4^lo^. Cells were analyzed using the FACSDiva (BD Biosciences). A representative image of the sorted cells are shown in Fig. S6.

### Western Blot

Whole cell extracts were isolated with M-PER. The extracts were assayed for total protein using Pierce BCA Protein Assay Kit (Thermo Scientific). Extracts (10 µg) were electrophoresed at 150 volts on gradient (4–20%) SDS PAGE pre-cast gels (Bio-Rad, Hercules, CA). The proteins were transferred onto PVDF membranes (Perkin Elmer, Waltham, MA) for 1 h with 100 volts at 4 °C. The membranes were incubated overnight with primary antibodies in 5% milk-PBS solution at 4 °C at 1/1000 final dilution. The same membranes were stripped with Restore Western blot Stripping Buffer and then reprobed with the different antibodies. The band densities were normalized to those of β-actin.

After incubation with the antibodies the membranes were washed four times with 1 × PBS containing 0.1% TWEEN. The membranes were incubated with HRP-anti-murine IgG (1/2000) or HRP-anti-rabbit IgG (1/2000), depending on the primary antibody for 2 h at 4 °C. The normalization antibodies for Histone H3 and β-actin were added at final dilution of 1/1000 and 1/4000, respectively. After this, the membranes were subjected to additional washes three times, followed by detection with Western Lightning Chemiluminescent Reagent. The molecular weights of the bands were determined by comparing with Kaleidoscope Pre-stained standards (BioRad). Band densities were normalized to β-actin with the Un-Scan-It software (Silk Scientific Inc, Orem, Utah).

### Flow cytometry

BCCs (5 × 10^6^) (MDA-MB-231 and T47D) with stable transfection of Oct4-GFP cells were resuspended in 2 ml of 1 × phosphate buffered saline (PBS, pH 7.4) and then fixed with 1 ml of 3.7% formaldehyde (diluted in PBS). Cells were incubated for 15 min at room temperature and then permeabilized for 30 min with 1 ml of 0.2% Triton-X 100 (diluted in PBS) at room temperature. Cells were washed twice with 2 ml of 0.5% Bovine Serum Albumin in PBS (incubation buffer) and then resuspended at 1:1 ratio of methanol:PBS. After 10 min incubation, cells were washed twice with incubation buffer. Cells were triple labeled for CD24 (1/30) and CD44 (1/30) and either for TMEM98 (1/100 final dilution), or FAT4 (1/200 final dilution) or GPR64 (1/200 final dilution), at 10^6^/150 μL of incubation buffer for 60 min at RT. After washing cells twice with incubation buffer, cells were stained with secondary antibodies at 1/500 final dilution for 30 min at RT in the dark. This was followed by two washes with incubation buffer. Cells were resuspended in 1% formaldehyde and immediately analyzed on the FACSCalibur (Becton and Dickson).

Mononuclear cells from the blood of patients were isolated by Ficoll Hypque Density Gradient. The cells were labeled for the markers listed above, expect for the addition of CD45, which was used to gate out the hematopoietic cells. In cases where there was sufficient cells, we included anti-pan-cytokeratin to gate these cells for analyses, and performed labelings for intracellular Oct4A.

Data were analyzed with BD CellQuest (BD Biosciences). The geometric (Geo) mean, shown in Fig. 4, was calculated by subtracting the mean fluorescence intensity (MFI) of isotype control from the experimental MFI. The relative amount of circulating tumor cells (CTCs) were determined by the relative MFI of specific subsets based on the hierarchy shown in Fig. 5.

### Aldefluor Assay

Aldefluor assay was performed with BCCs, untransfected or stably transfected with Oct4-dsRed cells using a kit from Stem Cell Technologies (Vancouver, Canada). The assay was performed according to the manufacturer’s instructions. The K562 cell line served as a positive control. The aldefluor reagent was added to the cells. After this, the reaction was stopped at different times with diethylaminobenzaldehyde (DEAB). The cells were immediately analyzed by flow cytometry. The untransfected BCCs were gated as the total population and the Oct4-dsRed transfectants were gated based on Oct4 level (red fluorescence). We used 15 milliwatt 488 nm laser. In the case of Aldeflour, we used bandpass 530/30 filter and for Ds-Red, 585/42 filter. We use single labeled controls to compensate spectral overlap to guide for further optimization.

### Telomere Length Assay

Telomere length was assayed with the *TeloTAGGG* Telomere Length Assay from Roche Diagnostics (Indianapolis, IN) according to the manufacturer’s protocol. The cells were sorted as described above and genomic DNA isolated with the QIAamp DNA Mini Kit from Qiagen (Valencia, CA).

### Real time PCR

RNA extraction was performed per manufacturer’s protocols with the RNeasy Mini Kit (Qiagen, Valencia, CA). Quality and concentration of RNA were determined with the Nanodrop ND-1000 spectrophotometer. The High-Capacity cDNA Reverse Transcription Kit (Life Technologies, Grand Island, NY) was used to convert RNA to cDNA. Real-time PCR was performed with 10 ng of cDNA using either Taqman Universal PCR Master Mix II or Power SYBR® Green PCR Master Mix (Life Technologies, Grand Island, NY). TaqMan primers were purchased for RAP1A and PPIB from Life Technologies, other primer sequences are in Supplemental Table 2.

### Affymetrix Array

MDA-MB-231-pEGFP1-Oct3/4 cells were sorted into three conditions as stated above. In four different experiments, total RNA was isolated from the sorted cells with Qiagen RNeasy Mini Kit (Qiagen). The RNA quality was assessed on the Agilent Bioanalyzer 2100. Total RNA (100 ng) was converted into cDNA, cRNA, and fragmented according to the manufacturer’s protocol with the assistance of the Biomek FX^P^ Target Prep Express with standard scripts for 3′ IVT Express for Gene Expression. Fragmented samples were hybridized to GeneChip Human Genome U133 Plus 2.0 Arrays and then washed and stained following the manufacturer’s protocols. The hybridized GeneChip Arrays were scanned with a GeneChip Scanner 3000 7 G (Agilent/Affymetrix) and the data saved in .cel files.

### Data Analyses

A CONSORT chart is outlined to show how the samples were used in the Affymetrix analyses (Fig. S1). Three BCC subsets were selected based on GFP expression, which we correlated with Oct4A expression, based on previous studies^3^. The sorted cells were assigned Oct4^hi^, Oct4^med^, Oct4^lo^. Oct4^hi^ and Oct4^lo^ were tested in quadruplicate, except for Oct4^med^, which was tested in duplicate. Genes < 1.2 fold among the different subsets were filtered and the resulting genes were subjected to further analyses with multiple programs. This allowed us to reduce inherent bias of the individual program: Partek^®^ Genomics Suite software, version 6.11.0801 (Partek Inc., St. Louis, MO), Gene Spring GX 10.0.2 (Agilent Technologies, Santa Clara, CA); QIAGEN’s Ingenuity® iReport (QIAGEN Redwood City, CA) and BRB-Array Tool, version 4.4.0 developed by Dr. Richard Simon and BRB-Array Tools Development Team. Oct4^hi^, Oct4^low^ and Oct4^med^ samples were considered as replicates if they were under the same condition. Average intensity values across each replicate were taken into consideration for analysis. Further statistical analysis was done in each program and noted in the figures.

Background corrected hybridization intensities were imported into BRB-Array Tools Version 4.4.0 log2-transformed and robust multi-array average (RMA) normalized (36). Features not significantly above background intensity in 50% or more of the samples, and features not changing at least 1.2-fold in at least 20% of the samples were filtered out. This yielded 25,750 features that were used in subsequent analyses.

BRB class comparison was conducted to identify genes that were differentially expressed. Two-sample t-test or F-test (more than 2 classes) based *p* value was calculated for each significant gene with random variance model. Genes with p-values of <0.001 were considered significant. The false discovery rate was also estimated for each gene to control false positive, as described^46^. Multidimensional scaling was performed in BRB-ArrayTools to represent high-dimensional data (e.g., a selected gene list with relative expression) graphically in low dimensions. The Euclidian distance metric was used to compute a distance matrix and the principal components of the gene expression signature. Each sample was then represented by a single point and the distance between two points indicated the overall similarity of those two samples. The first three principal components of gene expression were used as axes to generate a plot. Data was also visualized using hierarchical clustering in BRB-Array Tools. The Euclidean distance metric and average linkage was used to cluster genes and generate a heat map.

### Pathway and networking by Ingenuity Pathway Analysis (IPA)

The significantly differentially expressed genes (*p* < 0.001, FDR ≪0. 6%) were imported into Ingenuity Systems (http://www.ingenuity.com/). The IPA knowledge base filter mapped 2705 transcripts to known genes in IPA. We next performed IPA core-analysis in the context of pathways and networks, biological function and/or diseases. The right-tailed Fisher’s exact test was applied to calculate the *p* value ascertaining the probability that each biological function and/or disease. IPA core analysis identified 1721 genes as the highest significant functional category associated with cancer (*p* < 2.10E-15) and 344 genes relevant to BC (*p* < 3.10E-13). Of the 344 genes over-represented for BC, 99 of these genes have relative fold change >1.5. We used pathway analysis to determine whether Oct4^hi^ expression was connected to genes associated with BC at the molecular network level based on connectivity information in the IPA Knowledge Base. We added molecules suggested by the IPA “pathway explorer” in order to connect molecules of interest. Priority was given to those molecules with a high degree of connectivity within the pathway rather than molecules with many connections to molecules not on the pathway.

### Data availability

The dataset generated with the Affymetrix gene arrays are available through the GEO database using accession number GSE 86861.

## Electronic supplementary material

Supplemental information
