# Supplementary material for: Evaluation of a developmental hierarchy for breast cancer cells to assess risk-based patient selection for targeted treatment
Source: Sci Rep. 2018 Jan 10;8:367. doi: 10.1038/s41598-017-18834-5 (PMC5762675; doi:10.1038/s41598-017-18834-5)
Supplement: Supplementary file 1 — Supplemental information [file 41598_2017_18834_MOESM1_ESM.doc]

**SUPPLEMENTAL INFORMATION**

**Title:**  Evaluation of a developmental hierarchy for breast cancer cells to assess risk-based patient selection for targeted treatment.

**Authors:** Sarah A. Bliss, Sunirmal Paul, Piotr W. Pobiarzyn, Seda Ayer, Garima Sinha, Saumya Pant, Holly Hilton, Neha Sharma, Maria F. Cunha, Daniel J. Engelberth, Steven J. Greco, Margarette Bryan, Magdalena J, Kucia, Sham S. Kakar, Mariusz Z. Ratajczak, Pranela Rameshwar

Total RNA

Biological Replicates

Oct4^hi^

Oct4^low^

Oct4^med^

n=4 n=2 n=4

Filter genes with <1.2 fold changes

Data Analyses

**Supplemental Figure 1 (Fig. S1).** A CONSORT diagram of the method used to analyze RNA samples using Affymetrix.


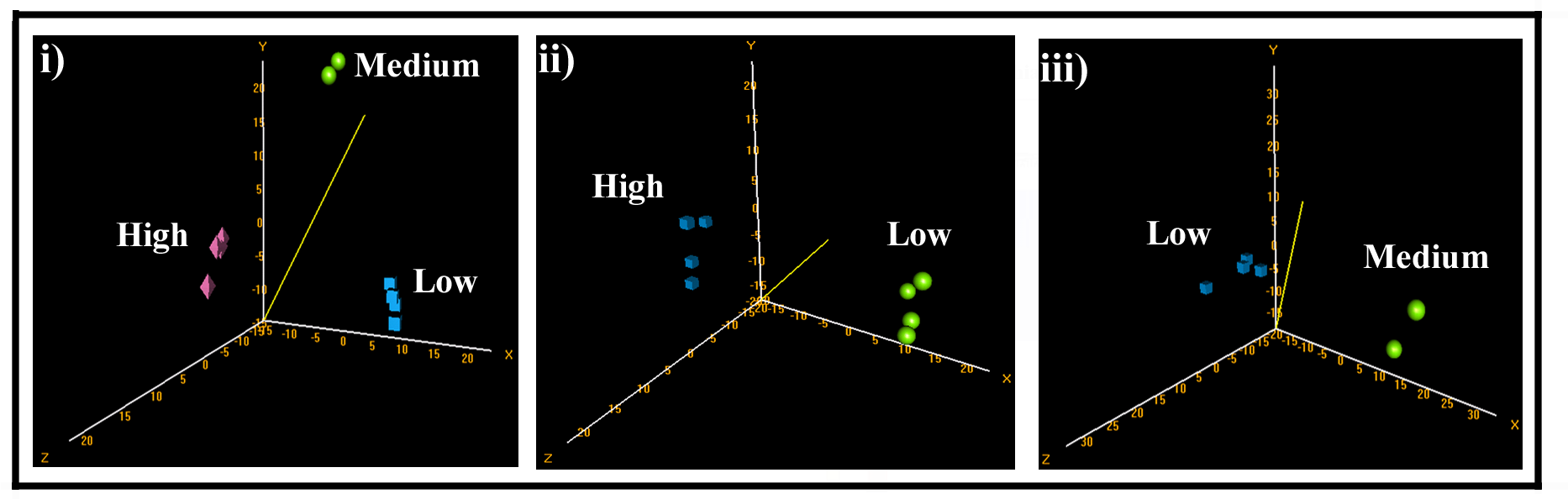


**Supplemental Figure 2 (Fig. S2).** (**i**) Multidimensional scaling (MDS) of all samples is shown to illustrate the separation of samples by their relative gene expression. Each point represents the relative expression of all 2227 genes differentially expressed at *p* < 0.001 and FDR <1.12%MDS. (**ii**) MDS comparing Oct4^hi^ and Oct4^lo^ conditions, each point represents the relative expression of all 2984 genes differentially expressed at *p* < 0.001 and FDR <0.86%. (**iii**) MDS comparing Oct4^med^ and Oct4^lo^ conditions, each point represents the relative expression of all 1675 genes differentially expressed at *p* < 0.001 and FDR < 1.53%.


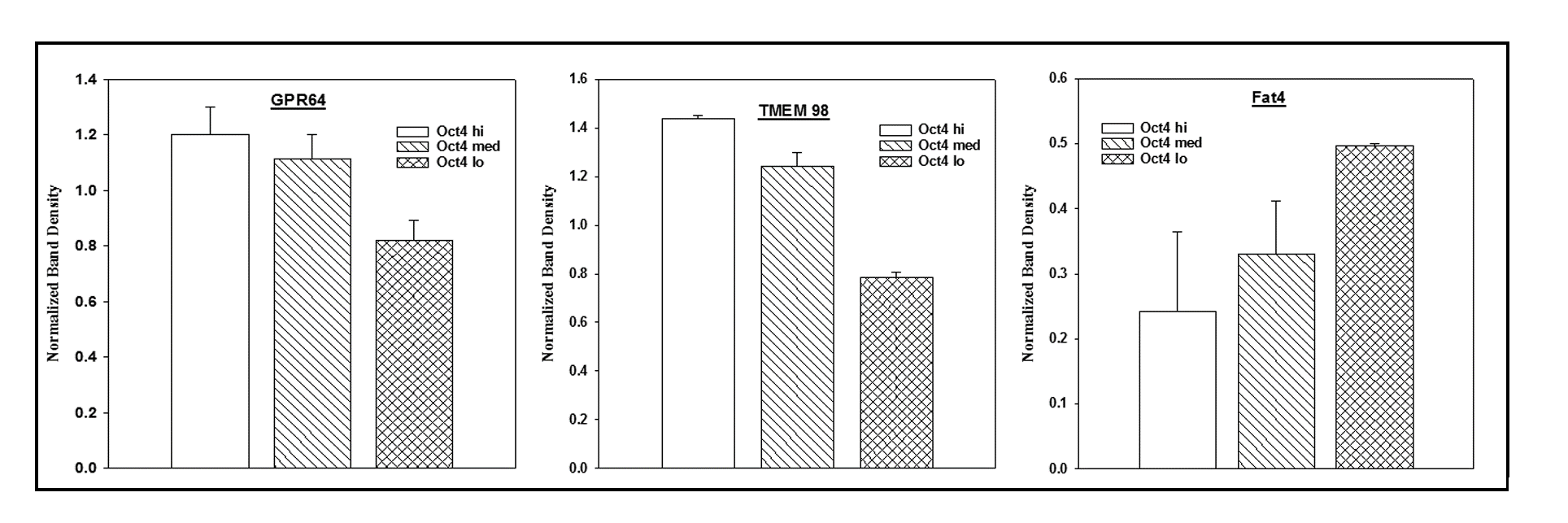


**Supplemental Figure 3 (Fig. S3).** Normalized densities of the bands for TMEM98, GPR64 and Fat4 from the western blot of Figure 2B. The bars are the mean±SD of five independent experiments.


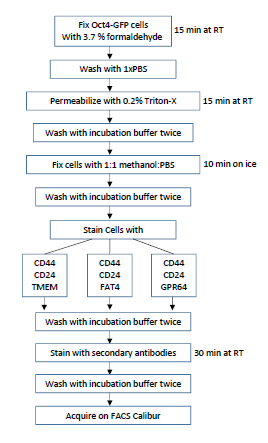


**Supplemental Figure 4 (Fig. S4).** The diagram describes how the cells were labeled for the markers used to stratify BCCs. ALDH1 was done separately using the kit described in Materials and Methods. The patient samples followed the same protocol as for the cell lines.


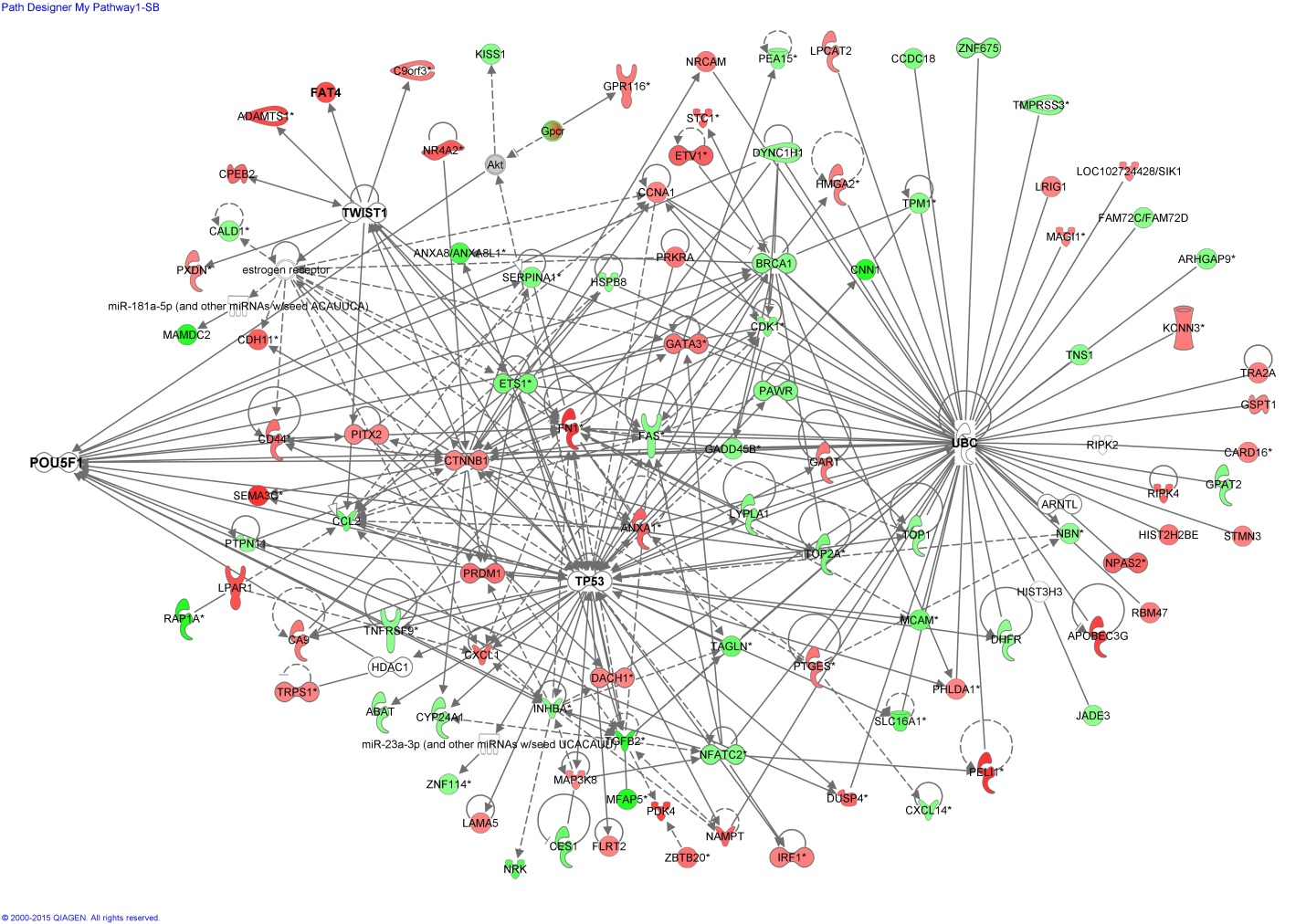


**Supplemental Figure 5 (Fig. S5).** The network was created with differentially expressed genes in BCCs, Oct4^hi^ and Oct4^lo^ using the following filtering criteria: 1.5 fold change and *p* value, <e^-9^.


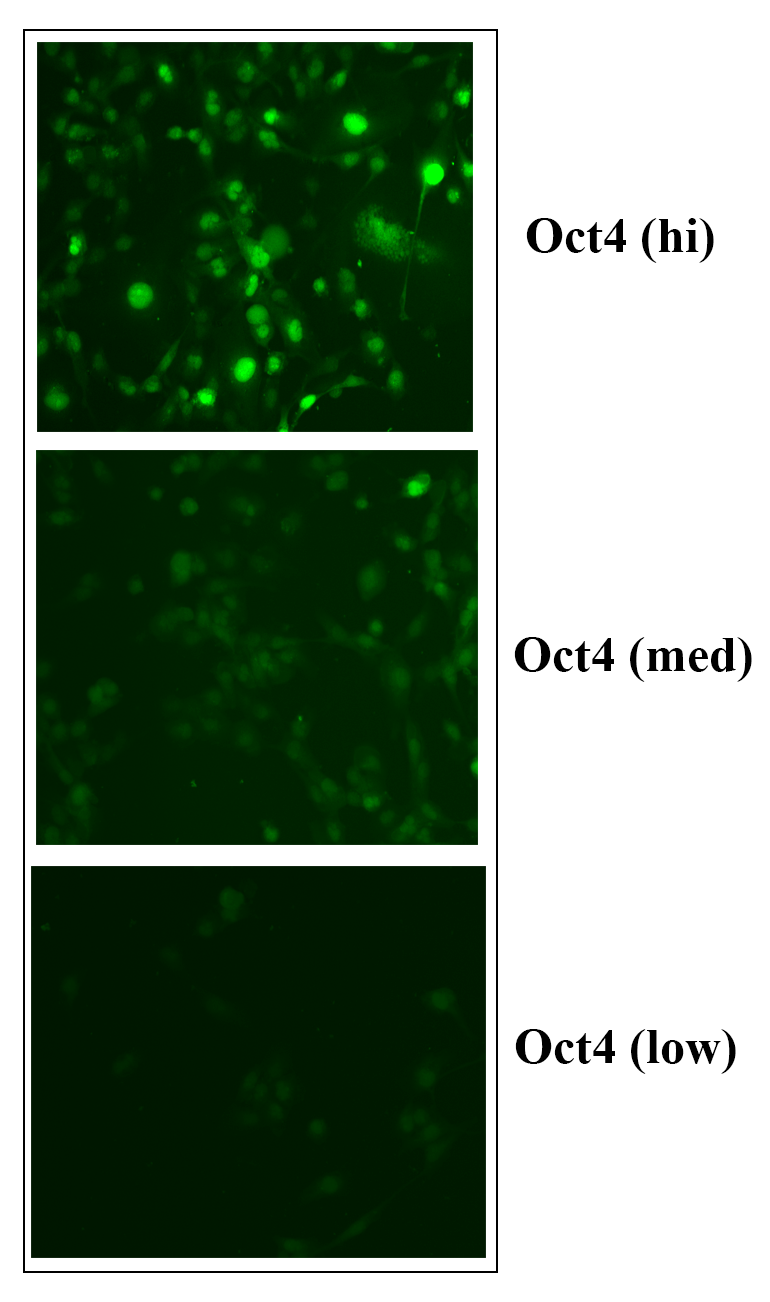


**Supplemental Figure 6 (Fig. S6).** The images were from cells immediately after sorting three subsets of Oct4A-GFP transfected MDA-MB-231.

**Supplemental Table 1 (Table S1): Relevant genes between Oct4^hi^ and Oct4^lo^ BCCs**

| **Symbol: Name** | **Fold Change** | **Location** |
| --- | --- | --- |
| RAP1A: RAP1A, member of RAS oncogene family | 21.96 | Cytoplasm |
| DAPK1: death-associated protein kinase 1 | 4.008 | Cytoplasm |
| CNN1: calponin 1, basic, smooth muscle | 3.299 | Cytoplasm |
| MAOA: monoamine oxidase A | 3.253 | Cytoplasm |
| **TMEM98: transmembrane protein 98** | 2.599 | Cytoplasm |
| STXBP6: syntaxin binding protein 6 (amisyn) | 2.33 | Cytoplasm |
| TAGLN: transgelin | 2.017 | Cytoplasm |
| FBXO32: F-box protein 32 | -2.031 | Cytoplasm |
| PADI4: peptidyl arginine deiminase, type IV | -3.412 | Cytoplasm |
| PDE4B: phosphodiesterase 4B, cAMP-specific | -3.85 | Cytoplasm |
| MFAP5: microfibrillar associated protein 5 | 3.944 | Extracellular Space |
| NPPB: natriuretic peptide B | 2.996 | Extracellular Space |
| MAMDC2: MAM domain containing 2 | 2.964 | Extracellular Space |
| EDIL3: EGF-like repeats and discoidin I-like domains 3 | -2.159 | Extracellular Space |
| FAM20C: family with sequence similarity 20, member C | -2.267 | Extracellular Space |
| HTRA1: HtrA serine peptidase 1 | -2.61 | Extracellular Space |
| FN1: fibronectin 1 | -2.801 | Extracellular Space |
| DHRS2: dehydrogenase/reductase (SDR family) member 2 | 7.818 | Nucleus |
| SOX4: SRY (sex determining region Y)-box 4 | -2.464 | Nucleus |
| ALPP: alkaline phosphatase, placental | 3.38 | Plasma Membrane |
| TENM2: teneurin transmembrane protein 2 | 2.977 | Plasma Membrane |
| **GPR64: G protein-coupled receptor 64** | 2.17 | Plasma Membrane |
| PDZD2: PDZ domain containing 2 | 1.942 | Plasma Membrane |
| TRIB2: tribbles homolog 2 (Drosophila) | -2.04 | Plasma Membrane |
| PLXNA1: plexin A1 | -2.151 | Plasma Membrane |
| SLCO4A1: solute carrier organic anion transporter family, member 4A1 | -2.158 | Plasma Membrane |
| **FAT4: FAT tumor suppressor homolog 4 (Drosophila)** | -2.193 | Plasma Membrane |
| SPRY1: sprouty homolog 1, antagonist of FGF signaling (Drosophila) | -2.417 | Plasma Membrane |
| KLRC2: killer cell lectin-like receptor subfamily C, member 2 | -4.647 | Plasma Membrane |
| FAM49A: family with sequence similarity 49, member A | 2.657 | Unknown |
| LOC645638: WDNM1-like pseudogene | 2.596 | Unknown |
| ECSCR: endothelial cell surface expressed chemotaxis and apoptosis regulator | 2.37 | Unknown |
| LOC100127888: uncharacterized LOC100127888 | -2.556 | Unknown |

The results for Table S2 were obtained from the analyses of Ingenuity iReport. The differences are statistically different.

**Supplemental Table 2 (Table S2).** Customized primer sequences for RT-PCR.

| **Primer (HGNC)** | **Forward Sequence (5′-3′)** | **Reverse Sequence (5′-3′)** |
| --- | --- | --- |
| GPR64 | TCA TTG TGG TCC TGG TTC | TTTCTTTGGCCACACAGT |
| FAT4 | c t t c c a a a t g g a c c c t g a g a | g g a a c t t c a c t a c c g g g t c a |
| TMEM98 | a g t c g a t g t a c c c t c c g t t g | c g a a g g a c t t c c a a a t g c t c |
| RAP1A | C A G G G C C A G A A T T T A G C A A G | A C T A T G G G C C T A G A G C A G C A |
| FAM49A | a g a t g a g g a a c c c g g c t a t t | t g g c a t t g c t a a g g g t t t t c |
| KLRC1 | t c c a t g g g t g a c a a t g a a t g | c t g c a a a t g c a a a c g c t t t a |
| KLRC2 | t t t c c c c g a a t a c a a g a a c g | c a c c a a t c c a t g a g g a a g g t |
| KLRC3 | c a t t t g c a t t g t c c t g a t g g | c a g g c c t g c a a a c t c t c t t c |
| FN1 | t g t t c g t g c a g c t g t t t a c c | g c c a c c g t a a g t c t g g g t t a |
| DUSP4 | c t g t g a c g g c a t c a t t c a t c | t g g a c t t c c a g g a a t c a a g g |
| ALOX5AP | g c g t t t g c t g g a c t g a t g t a | g a g a t g g t g g t g g a g a t c g t |

HGNC = Human Gene Nomenclature Committee-approved gene symbol.
